# The *KRÜPPEL*-Like Transcription Factor *DATILÓGRAFO* Is Required in Specific Cholinergic Neurons for Sexual Receptivity in *Drosophila* Females

**DOI:** 10.1371/journal.pbio.1001964

**Published:** 2014-10-07

**Authors:** Joseph Moeller Schinaman, Rachel Lynn Giesey, Claudia Mieko Mizutani, Tamas Lukacsovich, Rui Sousa-Neves

**Affiliations:** 1 Department of Biology, Case Western Reserve University, Cleveland, Ohio, United States of America; 2 Department of Genetics and Genome Sciences, Case Western Reserve University, Cleveland, Ohio, United States of America; 3 Department of Developmental & Cell Biology, School of Biological Sciences, University of California, Irvine, California, United States of America; The Rockefeller University, United States of America

## Abstract

Courtship is a widespread behavior in which one gender conveys to the other a series of cues about their species identity, gender, and suitability as mates. In many species, females decode these male displays and either accept or reject them. Despite the fact that courtship has been investigated for a long time, the genes and circuits that allow females to generate these mutually exclusive responses remain largely unknown. Here, we provide evidence that the Krüppel-like transcription factor *datilógrafo* (*dati*) is required for proper locomotion and courtship acceptance in adult Drosophila females. *dati* mutant females are completely unable to decode male courtship and almost invariably reject males. Molecular analyses reveal that *dati* is broadly expressed in the brain and its specific removal in excitatory cholinergic neurons recapitulates the female courtship behavioral phenotype but not the locomotor deficits, indicating that these are two separable functions. Clonal analyses in female brains identified three discrete foci where *dati* is required to generate acceptance. These include neurons around the antennal lobe, the lateral horn, and the posterior superior lateral protocerebrum. Together, these results show that *dati* is required to organize and maintain a relatively simple excitatory circuit in the brain that allows females to either accept or reject courting males.

## Abstract 2

Female decision-making in *Drosophila* flies requires the expression of a transcription factor in a small number of cholinergic neurons in discrete brain regions.

## Author Summary

Males of the fruit fly *Drosophila melanogaster* generate a series of courtship displays that convey visual, auditory, and olfactory information that females must decode in order to accept or reject mating. Despite the central role of female decision in sexual selection, relatively little is known about how genes and neural circuits generate this behavior. Here we show that the transcription factor *datilografo* (*dati*) is required to organize and maintain the neural circuitry required for acceptance in the central brain. Strikingly, *dati* is required in an excitatory circuit involving few neurons that express acetylcholine as their neurotransmitter and are located in the olfactory lobe, the first entry point for odor processing in the brain. In addition, *dati* is required in two other brain centers: a region where olfaction and presumably other senses are integrated and a novel region. Together these results show that a complex behavior can be generated by very few excitatory neurons, suggesting that the sharp cutoffs between acceptance and rejection may involve different thresholds of stimulation as postulated decades ago.

## Introduction

Animals are capable of a staggering array of complex behaviors and many of them rely on innate abilities to compare different scenarios and generate specific and appropriate responses. For instance, most animals can determine with ease whether the best option is to confront or retreat from a predator or opponent. Risk assessment and similar mutually exclusive behaviors are likely to rely on neural circuits that collect information, remove irrelevant and noisy information, and quickly determine a course of action.

Courtship rituals are ancient forms of communication that allow animals to identify and rank potential mates in the midst of a noisy and usually complex environment. Thus, it is not surprising that courtships usually deploy a series of displays that involve bright colors, unusual sounds, and rhythmicities. The recipients of these displays, which in many species are females, evaluate their quality and generate the mutually exclusive behaviors of accepting or rejecting courtship.

One of the most fascinating aspects of the ability to generate courtship and respond with a decision is the fact that both behaviors are largely genetically encoded; that is, animals are capable of executing them perfectly with minimal practice and no instruction every generation. Pioneering work has established clear associations between individual male courtship behaviors with specific genes and alleles in Drosophila [1], and even led to the mapping of foci in the central nervous system required to generate discrete behaviors [2]–[5]. However, little is known about how females interpret and integrate aspects of the male's displays and decide if and when to accept male courtship [6]. This is a longstanding question of significance not only to our understanding of the molecular mechanisms of reproductive behavior but also to any comprehensive understanding of how neural circuits generate mutually exclusive decisions.

In Drosophila, males show their interest in females by making wing displays, singing a courtship song, dispersing airborne and contact pheromones, and physically contacting them [7]–[9]. In response to these cues, receptive females slow their movement and allow the male to proceed, to finally posture themselves to allow the male to mount them for copulation. In contrast, a disinterested or unreceptive female will engage in a number of rejection behaviors, such as fleeing, kicking the male, extruding her ovipositor, and raising or curling her abdomen [7]. Early studies have shown that no single sensory modality alone determines acceptance or rejection in mature females. Instead, the likelihood of acceptance or rejection relies on different sensory modalities that individually contribute to the final behavioral output [10]–[13]. Genes and alleles that either enhance or inhibit female receptivity have been isolated [14]–[17]. Mutations in these genes provide a unique opportunity to determine the genetic contribution to cell organization and physiological responses required to generate female mate choice [18]. In addition to mutations, somatic mosaics have been employed to determine the regions of the brain underlying female behavior [18],[19]. Nevertheless, critical information about the neural circuitry involved in female decision-making behavior and the genes that pattern these circuits is still sorely lacking. Here, we describe *dati*, a neural-specific transcription factor that is required for female courtship acceptance and locomotion, and use it to begin probing the nature of the circuit by which females integrate the signals they receive from courting males to reach the correct behavioral output.

## Materials and Methods

### Mutants, Transgenes, Genotypes, and Stocks

Flies were reared at 25°C or 22°C on standard cornmeal–molasses–yeast media (http://flystocks.bio.indiana.edu/Fly_Work/media-recipes/molassesfood.htm). The descriptions of fly stocks used and mutations therein can be found at (http://flybase.bio.indiana.edu/), unless otherwise stated. The mutant *l(4)102CDd* ^2^ [20] was molecularly mapped in this study. The single breakpoints of the chromosomal deletions *Df(4)C1-7A*, *Df(4)B6-4A*, and *Df(4)B6-2D* and the compound chromosome C(4)DRA-1 were described previously [21],[22]. The mutations *KG02689* (*dati* ^1^) and *KG01667* (*dati* ^2^) were also described previously [22],[23] and correspond to single *P* element insertions in the second intron and 300 bp upstream of the first exon of *CG2052*, respectively. *dati* ^F11.4^ was generated by the excision of the *P-element KG02689* inserted in *dati* ^1^ and corresponds to a precise excision as revealed by direct sequencing of the region that flanks the insertion. UAS-*dati* ^F32D^ is a leaky UAS transgene containing the cDNA of *dati* (GH06573, *CG2052*-PA) that rescues the embryonic lethality of *l(4)102CDd* ^2^ up to the first instar in the absence of a driver and its insertion was determined to be on the second chromosome in the neurally expressed gene *Mmp2*. The *D. melanogaster* stocks used were as follows: wild-type Canton-S (CS), *w* ^1118^, *y w*, *y w*; Df(4)B6-4A/*ci* ^D^ *spa^p^* ^ol^, *y w*; Df(4)B6-2D/*ci* ^D^ *spa^p^* ^ol^, *y w*; Df(4)C1-7A/C(4)DRA-1, *y w*; Df(4)BH/C(4)DRA-1, *y w*; C(4)DRA-1/*dati^1^*, *y w*; *dati^F11.4^*, *y w*; SM/*Cha*-Gal4 UAS-GFP; C(4)DRA-1/*dati^1^*, *y w* hs-FLP; SM/FRT42D *Actin*-Gal4; *ci* ^D^ *spa*/*dati* ^1^, *y w* hs-FLP; SM/FYGal80T; *spa* ^pol^ [23], *w*; *UAS-mCherry.NLS*, *w*; *dati* RNAi/*dati* RNAi [FBst0472372], *w UAS-dcr-2*; dati RNAi, *y w hs-FLP*; *SM*/FRT42D *Actin*-Gal4; C(4)DRA-1/*dati* ^1^, *elav*-Gal4 [FBst0008760], *w*; *TM3*, *Sb*/*repo*-Gal4 [FBst0007415], *w*; CyO/*Cha*-Gal4 UAS-GFP [FBst0006793], *w*; *ple*-Gal4 [FBst0008848], *w*; *Ddc*-Gal4 [FBst0007009], *w*; *Gad*-Gal4 (gift of T. Sakai) [17], Ubi-GFP.NLS [FBst0005626], Ubi-RFP.NLS [FBst0035496], and FYT/GAF [23].

Experimental crosses were raised at 25°C, unless otherwise specified. Both males and females used for mating experiments were collected as pupae and aged 3–6 d posteclosion before mating tests. All mating tests were performed at 22°C, between 1 and 4 pm EST.

### Recording of Mating Behavior

Mating tests were performed in small arenas made by superimposing two sliding sheets of transparent polycarbonate containing 24 wells each (2.54 cm in diameter and 1.27 cm depth) [24]. Each well was divided in half by a thin removable sheet of plastic. Canton-S males and experimental females 3 to 6 d old were loaded into opposing sides of each chamber without anesthesia with a manual aspirator. Once all wells were loaded, the thin plastic sheet was removed and all pair matings began simultaneously. The chamber was lit from below by an Artograph LED LightPad (Artograph Inc.), and mating behavior was recorded using a Sanyo FH1-A (Sanyo Inc.) camcorder for 1 h. For each experimental group, we calculated the courtship acceptance rate, defined as the number of pairs that successfully copulated in the 1 h observation period divided by the number of pairs observed. The average Courtship Index (CI) was calculated for each experimental group. CI is defined as the fraction of time a male spent courting in a given observation period [25]. Male courtship for each pairing was observed for 10 min, starting at the onset of courtship. CIs for each pair mating in an experimental group were then aggregated into an average CI. Sample sizes are shown in the corresponding figure in results.

### Quantification of Discrete Female Behaviors

Females 3 to 5 d old of experimental and control genotypes were pair-mated to Canton-S males in the mating chamber described above and video recorded for 1 h. For each pair mating, female behavior was analyzed for 10 min from the onset of courtship or until mating occurred. For this time period, every time a male initiated a step of courtship, the female reaction to courtship was recorded. The following six discrete rejection behaviors were quantified: fleeing, kicking, extruding ovipositor, jumping, flicking wings, and standing still. For each female, a Behavioral Index (BI) was obtained by calculating the frequency of each behavior displayed over the frequency of all behaviors, and these indices were averaged for each genotype.

### Analysis of Locomotor Behavior

Locomotor behavior was analyzed using an adaptation of the negative geotaxis assay [26]. Five to eight flies of 3 to 5 d old were placed in a 15 mL Falcon tube without anesthesia and allowed to acclimate for 5 min. After this period of acclimation, the Falcon tube was inverted and rapped sharply against a fly transfer pad three times to knock flies to the bottom of the tube. The tube was then placed in front of the camcorder and flies were allowed to climb the walls. The heights reached by each of the flies after 5 s was assessed from the camcorder footage. Over 30 flies were analyzed for each experimental group. For each Falcon tube of flies, this assay was repeated for a total of five trials, spaced 30 s apart, and the heights of all flies from each trial averaged together.

### Antibodies and Immunostaining

Antibody staining for brains was performed according to standard protocols [27]. Primary antibodies used were chicken anti-GFP (1∶1,000, Invitrogen), rabbit anti-RFP (1∶1,000, Invitrogen), mouse anti-nc82 (1∶1,000, DSHB), Guinea pig anti-Dati (1∶1,000, gift from T. Isshiki [35]), rat anti-Elav (1∶1,000, DSHB), and mouse anti-FasII (1∶100, DSHB). Secondary antibodies used were donkey anti-mouse 647 (1∶500), goat anti-rat 555 (1∶500, Invitrogen), donkey anti-guinea pig 647 (1∶500, Invitrogen), donkey anti-chicken 488 (1∶500, DyLight), and donkey anti-rabbit 555 (1∶500, Invitrogen). All samples were mounted in SlowFade (Invitrogen) and scanned on a Zeiss LSM 700 confocal microscope. Images generated from Z-stacks taken at 1 or 2 µm intervals are displayed as maximum intensity projections using Zeiss Zen 2009 or as orthogonal projections/surface projections using Image J.

### Automated Image Analysis and Cell Counting

Automated cell counting was performed on confocal slices using Fiji software [28]. Briefly, a two-channel stack stained for *dati* (green) and *Cha*-Gal4 UAS-RFP.NLS (red) was converted to RGB, and the yellow overlap was segmented with white color using “Threshold Color” function. The blue channel containing the segmented nuclear overlaps was extracted and the noise removed by filtering the stack with the function “Despeckle.” Three-dimensional segmentation counts were generated by the plugin “3D Object Counter” [29]. Due to the large size of posterior brain stacks, they were stitched together using the plugin “Pairwise Stitching” before segmentation [30].

### Clonal Analyses

Clonal analyses were performed using the FYT (FLP-recombinase recognition target site-*yellow^+^*-Translocation) system previously described [23]. After clone induction, third instar larvae containing GFP+ clones were handpicked, placed in a single vial, and allowed to develop up to adults 3 to 6 d old. Single females carrying clones were tested with single Canton-S male in the courtship arenas described above and video recorded for 1 h. After this time the number of couples that mated was recorded and the Courtship Indices determined. In the next day, the females that rejected males were retested with new Canton-S males for rejection, and only those that passed in the double rejection test were analyzed further [19]. Females that accepted and rejected males were referenced to specific wells and had their brains dissected. Each clone was located in a grid that divides the brain in 40 anterior and 40 posterior sectors. Because each brain may vary slightly in size or in the way it is mounted, the grid was manually stretched to find the best fit for each sample. In total, 491 clones from 83 brains were analyzed.

### Olfactory Behavior Assays

In these experiments, we used a T-Maze [31] with 2 µl of benzaldehyde in one of the ends. Individual flies were loaded into the elevator of the apparatus and immediately lowered to the level containing the two ends with and without odor. After 10 s, the number of flies that moved away from the aversive odorant or towards it was recorded.

### Mushroom Body and Antennal Lobe Image Analysis

Brains of different genotypes were dissected and stained for the mushroom body marker FasII and imaged as z-stacks at 1 µm intervals. Selected z-stacks containing the gamma lobe were manually segmented using the Fiji plugin “Segmentation Editor.” Measurement of γ lobe morphological defects was done in Fiji. A similar procedure was done to segment the antennal lobe, except that the limits of the segmented structures were defined by the expression of GFP in the pattern of CHA.

### Statistical Analysis

All statistical analyses were performed using MiniTab 16.1.0 (Minitab Inc.). For all comparisons of courtship acceptance rate between control and experimental groups, a 2-Proportion Test was performed, and Fisher's exact *p* test value was used for the determination of significance level between two groups, unless otherwise indicated. CI data were arcsine transformed prior to statistical analysis as previously described [32] and analyzed by one-way ANOVA. The difference in climbing ability in locomotor tests was analyzed by one-way ANOVA. Behavioral indices (Figure S2) were analyzed by Mann–Whitney U Test. All other tests are two sample *t* tests unless otherwise noted.

## Results

### Identification of *dati* on the *Drosophila* Fourth Chromosome

We previously generated a series of molecularly mapped terminal deletions on the fourth chromosome that define relatively small genomic intervals that can be used to map mutations [22],[33]. These deletions were then used to map a collection of mutants available for this chromosome, to later test for locomotion and other behavioral abnormalities. One of them was *l(4)102CDd^2^*, an unmapped embryonic lethal mutation isolated nearly 50 years ago by Ben Hochman [20].

**Figure 1:**
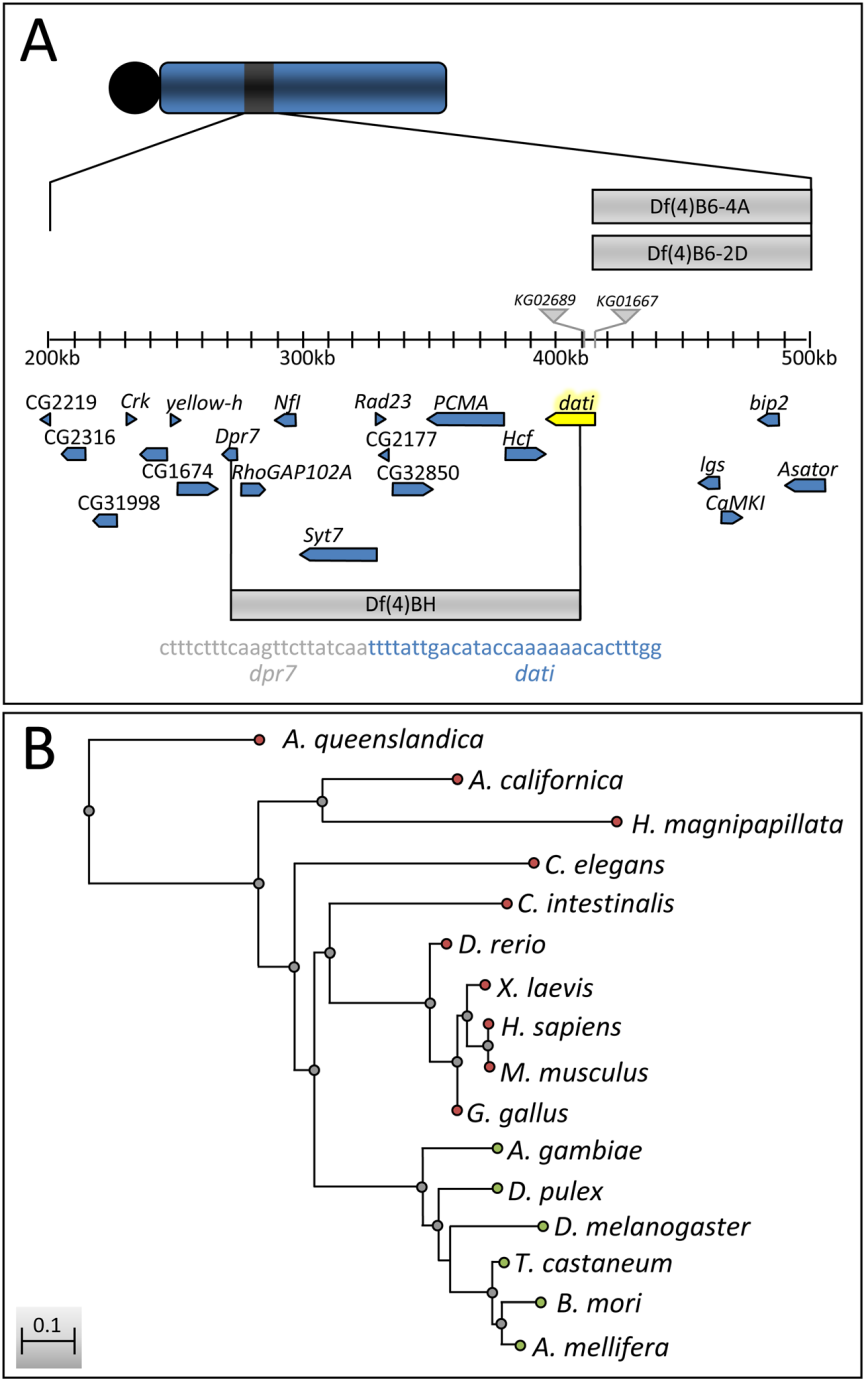
Molecular mapping of *dati^1^* and conservation of *dati* across species. (A) Physical position of *dati* on the fourth chromosome (highlighted in yellow). The *P*-elements *KG02689* (*dati* ^1^) and *KG01667* (*dati* ^2^) are represented by grey triangles. Deficiencies causing lethality in conjunction with *dati^1^* are represented by gray bars. Note that the *Deficiency of Ben Hochman* [*Df(4)BH*] spans a region of 10 genes, between *dpr7* and *dati*. Indicated below Df(4)BH is the sequence of the breakpoint in *dpr7* (gray) and *dati* (blue). (B) Neighbor-joining distance tree with Kimura two-parameter distances of *dati* sequences across multiple species. Branches of the tree with green termini represent orthologs of *dati*, whereas branches with red termini represent homologs.

While mapping *l(4)102CDd^2^* we found that 5%–8% of the heterozygotes between this mutation and two deletions (*Df(4)B6-2D* and *Df(4)B6-4A*) escaped the lethality of *l(4)102CDd^2^* and exhibited a phenotype of uncoordinated movements, which becomes stronger with age (compare Movies S2 and S3). Due to the tapping of the forelegs of these genotypes, we named the mutation *datilógrafo* (*dati*) [34], which means typist in Portuguese. Subsequent analyses revealed that mutations in *dati* also render females completely unable to accept male courtship, as will be shown later. We located molecularly the mutation in *l(4)102CDd^2^*, which corresponds to a deletion that disrupts *dpr7* and *CG2052* plus eight other genes in between, and renamed it as *deficiency on the fourth chromosome of Ben Hochman* [*Df(4)BH*] (Figure 1A and Text S1). Because two single fourth chromosome *P-element* insertions localized at the breakpoint of these deletions in the *CG2052* gene (*KG02689* and *KG01667*) exhibited the same phenotypes as homozygotes or heterozygotes for *Df(4)BH*, we focused our analyses on the insertion *KG02689* (*dati* ^1^), the strongest of these two alleles. *dati* encodes a zinc finger transcription factor closely related to *rotund* (*rn*) and *squeeze* (*sqz*) with homologs in several species, including humans (Figure 1B).

Consistent with its reported requirement in specifying late born neurons during embryogenesis [35], *dati* is specifically expressed in the central nervous system in embryos (Figure S1A). In larval stages, *dati* is expressed in the brain and ventral nerve cord (Figure S1B) but not in other larval tissues (e.g., wing, leg, eye, and antennal discs; unpublished data). In adults, *dati* is broadly expressed in the brain (Figure S1C).

### *dati* Mutant Females Are Courted Normally But Fail to Accept Male Courtship

*dati* ^1^ mutants usually stand still for long periods of time, but when courted by males, they can flee at considerable speed. In addition, when cornered by a courting male, they engage in a series of rejection behaviors that include kicking and curling their abdomen (Movies S1, S2, S3) [7],[36]. To investigate how the behavior of *dati* ^1^ females departs from the wild type, we quantified six discrete behaviors normally displayed by wild-type females in response to male courtship (i.e., fleeing, kicking, extruding ovopositor, jumping, flicking wings, and standing still). *dati* females display all of the aforementioned behaviors but spend more time kicking and less time standing still than the wild type (Figure S2).

**Figure 2:**
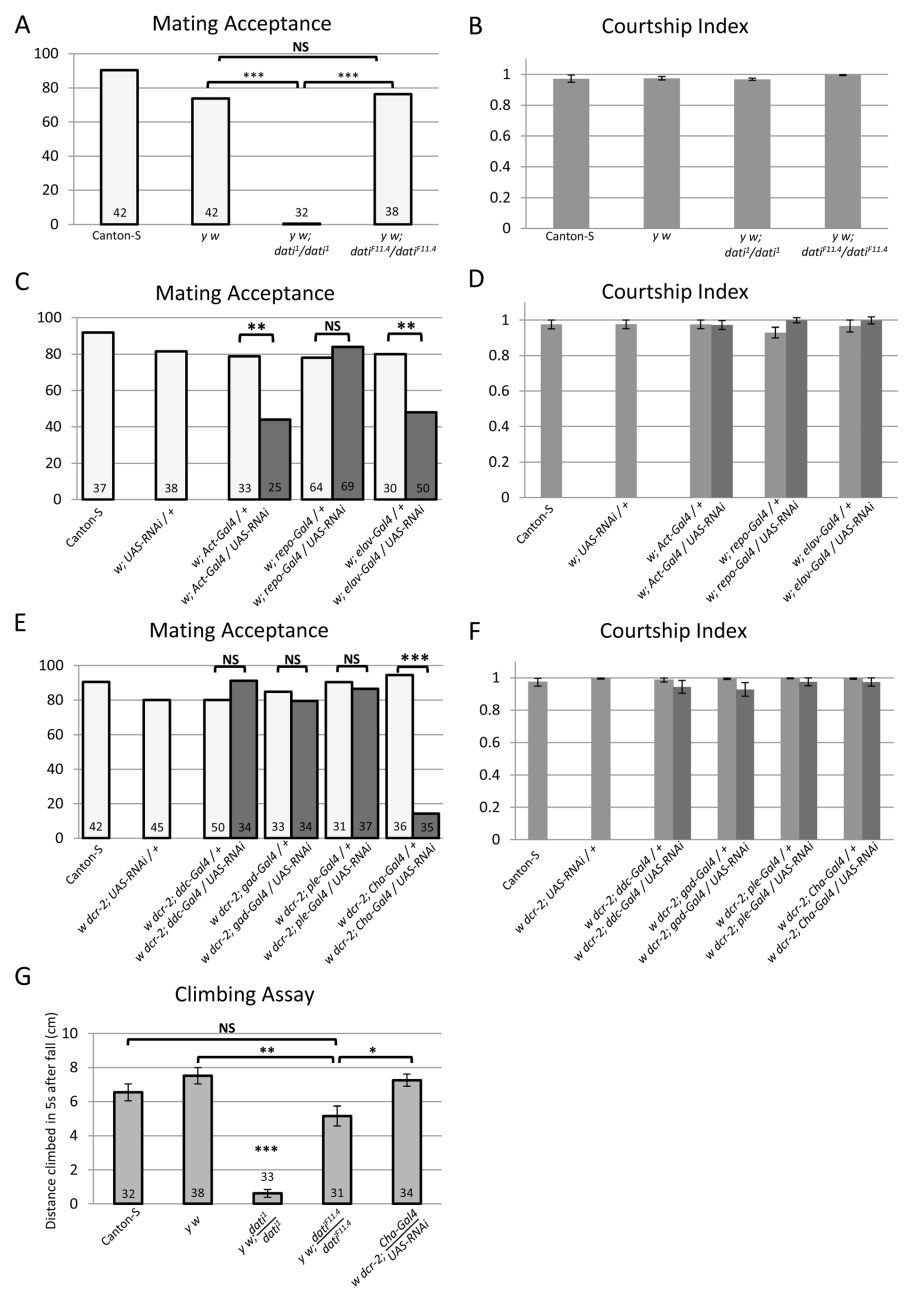
Results of pair mating and locomotor experiments using *dati^1^* and UAS-*dati*-RNAi. (A, C, E) Percentage of females that accepted wild-type Canton-S male courtship within 1 h in pair mating assays. (B, D, F) Courtship indices of Canton-S males towards females tested in (A), (C), and (E), respectively. Courtship indices were arcsin transformed and subject to one-way ANOVA, and in all cases they are not significantly different from each other (*p*>.05). Error bars represent ±SEM of the measurements of each genotype. (A–F) Genotypes of females tested are indicated (“UAS-RNAi” means UAS-*dati*-RNAi). Dark and light bars in (C–F) indicate experimentals and controls, respectively. (A, B) *dati* ^1^ homozygous females strongly reject males, despite being courted vigorously by Canton-S males. This rejection phenotype is reverted in revertant females homozygous for the precise excision *dati^F11.4^* allele. Controls with Canton-S and *y w* females were used, as revertants lose both *y*+ and *w*+ transgenes. (C, D) Ubiquitous expression of UAS-*dati*-RNAi driven by *Act*-Gal4 or expression in postmitotic neurons driven by *elav*-Gal4 causes rejection of male courtship. (C) Dark bars indicate the percentage of females expressing UAS-*dati*-RNAi driven by different Gal4 drivers that accepted male courtship. Light bars indicate results with control females (Canton-S females and females with either UAS-*dati*-RNAi or a Gal4 driver). Expression of *dati*-RNAi in glial cells (*repo*-Gal4) does not cause rejection. (E, F) Expression of *dati*-RNAi specifically in cholinergic neurons causes the female rejection behavior. (E) Percentage of courtship acceptance in females expressing UAS-*dati*-RNAi driven by neuron-specific Gal4 drivers (dark bars) versus control females with either Gal4 drivers or UAS-*dati*-RNAi only (light bars). *Ddc*-Gal4 is a driver of both serotonergic and dopaminergic neurons, *ple*-Gal4 of dopaminergic neurons, *Gad*-Gal4 of GABAergic neurons, and *Cha*-Gal4 of cholinergic neurons. (G) Courtship acceptance and locomotor deficits in *dati* mutant females are separable phenotypes. The graph shows the distance climbed by females of various genotypes within 5 s after being knocked to the bottom of a vial. Measurements shown are an average of five replicates from each group; error bars represent the mean ± the standard deviation. Sample size numbers are indicated inside bars in (A, D, E, G). Graphs (B, D, F) use the same datasets as (A, C, E), respectively. Statistical significance of differences in (A, C, E, G) was evaluated by the Fisher's exact probability test (***p<0.001; **p<0.01; *p<0.05).

To further quantify the abnormal mating behavior of *dati* ^1^ mutant females, we compared their mating success with that of wild-type Canton-S, *y w*, and *dati* precise excision revertant females. From these data it becomes evident that the behavior of *dati^1^* is significantly different from the wild-type Canton-S, *y w* and the revertant *dati^F11.4^* females, which exhibit normal acceptance rates (Figure 2A). To test whether the deficit in matings was exclusively due to the female rejection, we assessed the sex appeal of *dati* ^1^ homozygous females using the CI (Figure 2B) [25],[37]. These experiments reveal that males respond to *dati^1^* females normally, with courtship indices indistinguishable between all four groups.

The rejection of *dati* mutants was tested over a longer time by measuring the frequency of females that produced progeny with a wild-type male in 6 d. The difference between the two groups is not significant (*dati* ^1^ 6 d = 2/14 versus *dati* ^1^ 1 h = 0/32, *p* = 0.08), but both are significantly different than Canton-S (*dati* ^1^ 6 d versus Canton-S 6 d = 29/30, *p*<0.0001 and Figure 2A). This result is consistent with the fact that females that fail to accept males within 30 min are unlikely to mate afterwards [38].

### *dati* Is Required In Neurons for Normal Acceptance and Locomotion

To determine in which tissues *dati* is required for normal courtship behavior and locomotion, we knocked down its expression using RNAi and *UAS-dcr-2* to enhance the knockdown. The knockdown of *dati* with the ubiquitous *Actin*-Gal4 [23] at 25°C resulted in few adult individuals that died shortly after eclosion with extreme locomotor abnormalities (unpublished data). To obtain a less severe phenotype more similar to *dati* ^1^ homozygotes, the *UAS-dcr-2* construct was removed from the genotype and the flies were reared at 18°C. Under these conditions, females expressing the *dati* RNAi from *Actin*-Gal4 showed defects in acceptance and locomotion (Figure 2C,D; unpublished data). Similarly, the knockdown of *dati* with *elav*-Gal4 caused rejection and locomotor defects (Figure 2C). *elav* is a bona fide postmitotic marker, except for a transient embryonic expression in glial cells and neuroblasts in thoracic and abdominal segments [39]. However, we show that the knockdown of *dati* in glial cells using *repo*-Gal4 produced no effect (Figure 2), indicating that the courtship behavioral phenotypes are not generated in these cells. In addition, we later provide evidence that the behavioral effects of *dati* knockdown with *elav*-Gal4 are not associated with neuroblasts of the embryonic ventral nerve cord.

### The Removal of *dati* in Cholinergic Neurons Impairs Normal Female Acceptance But Not Locomotion

Because our previous results suggested that *dati* might be required in some capacity in neurons, we next asked whether a specific neuronal population could phenocopy the mating deficit observed. The fly brain employs several neurotransmitters including dopamine, acetylcholine, GABA, glutamate, serotonin, histamine, octopamine, and tyramine [40]–[45]. To begin an unbiased search for specific neuronal populations, we first knocked down the expression of *dati* by RNAi using four Gal4 drivers of genes involved in the synthesis of different neurotransmitters (*Dopa decarboxylase*, *pale*, *Choline Acetyltransferase*, and *Glutamic acid decarboxylase 1*) (Figure 2E,F) to later test other neuronal types if necessary. Out of the four drivers tested, Choline Acetyltransferase Gal4 (*Cha*-Gal4) produced a strong and significant reduction in courtship acceptance (Figure 2E,F). Thus, the inability of *dati* females to accept males affects a particular neuronal type.

Interestingly, the removal of *dati* in cholinergic neurons does not impair locomotion as can be observed from “negative geotaxis” escape response tests [26],[46]. In these tests, *dati* ^1^ homozygous females normally achieve a much lower mean height 5 s after being knocked to the ground compared to wild-type Canton-S females (Figure 2G). Revertants also have a significantly better climbing ability than *dati* homozygotes (Figure 2G). However, their climbing ability was not completely restored to the levels of *y w*, indicating that although most of the climbing deficits can be ascribed to the mutation in *dati*, other genes in the genetic background contribute to the locomotor deficits observed. In contrast, the climbing abilities of *dati* RNAi knockdowns with the *Cha*-Gal4 driver were not different from wild-type Canton-S (Figure 2G), indicating that the male rejection behavior of *dati* ^1^ mutants is separable from the locomotor deficits.

### *dati* Mutants Exhibit Abnormal γ-Lobes of the Mushroom Bodies, But These Defects Do Not Cause Female Rejection

**Figure 3:**
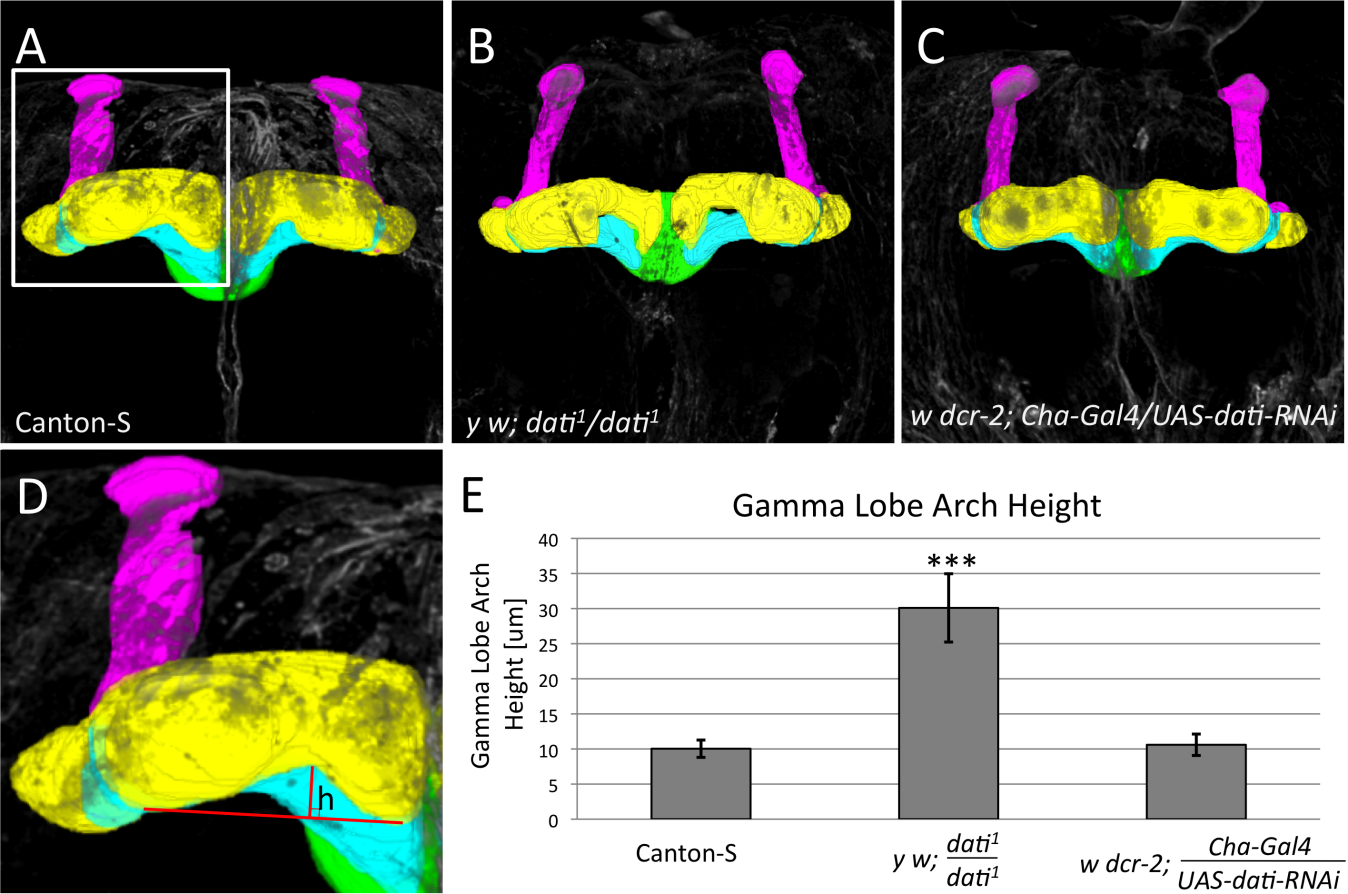
*dati* ^1^ homozygote females exhibit mushroom body defects, whereas *Cha*-Gal4/*UAS-dati*-RNAi females do not. (A–D) 3D segmentations of mushroom bodies visualized by anti-FasII staining of (A) wild type, (B) *dati^1^* homozygous, and (C) *Cha*-Gal4/*UAS-dati-RNAi* females. The α lobe (magenta), β lobe (blue), γ lobe (yellow), and ellipsoid body (green) are indicated. Note that the γ lobes in (B) are more curved than the gamma lobes in (A) and (C). Note that the genotype in (C) causes rejection like *dati^1^* homozygous females (B), but not locomotor defects (Figure 1G). (D) Mushroom body shown in (A, box) and measurement of the morphological defect of the γ lobe. Red lines show the base (horizontal) and height (vertical) of the mushroom body arch. (E) Graph shows that there is a significant increase in the height (h) of the mushroom body arch in comparison to wild-type and *Cha*-Gal4<UAS-*dati*-RNAi females. All images are 3D reconstructions of confocal stacks rendered in Fiji. Error bars are ±standard deviation. Significance was tested by a two-sample *t* test and *** indicates *p*<0.001. The 3D reconstructions of the mushroom bodies are shown in Movie S4. Sample sizes are: Canton-S (*N* = 12), *dati* ^1^ (*N* = 12), and *Cha*-Gal4>UAS-*dati*-RNAi (*N* = 14).

The results above revealed that the acceptance deficits of the *dati* ^1^ mutant are generated in cholinergic neurons. Because the mushroom bodies in *Drosophila* express CHA and have been implicated in memory formation, learning, and olfactory processing, we initially tested whether this neuropile was abnormal in *dati* ^1^ mutants [47],[48]. The alpha and beta lobes of *dati* ^1^ mutants appear indistinguishable from the wild-type mushroom bodies, but the gamma lobes are malformed with a generally withered appearance (Figure 3A,B,D) and have significantly different curvature (Figure 3E). To determine if the gamma lobe defects could be responsible for the behavioral rejection, we asked whether the knockdown of *dati* expression in CHA+ cells could recapitulate the morphological defect in the gamma lobe and behavioral phenotypes observed. These experiments revealed that although *Cha*-Gal4 *UAS-dati-RNAi* females reject males, the gamma lobe is not affected (Figure 3C,E). Together these experiments allowed us to conclude that although the loss of *dati* disrupts the gamma lobe neuropile, the focus of *dati*-mediated courtship acceptance lies elsewhere in the brain.

### *dati* Is Expressed in a Large Set of Neurons But in a Small Subset of the Cholinergic Neurons

To narrow the region where *dati* is required for female acceptance, we asked whether DATI- and CHA-positive neurons corresponded to a smaller subset than CHA neurons. DATI is broadly expressed in a complex pattern that involves a few thousand neurons. Automated cell counts indicate that there are around 2,400 neurons of the central brain that express *dati*, which corresponds roughly to 6.6% of all neurons of the fly's central brain (Figure S3) [49]. The overlap between CHA- and DATI-positive neurons is much smaller, comprising 345±55.3 (mean ± s.d) neurons of the anterior central brain (*N* = 5), and 1,049±134 neurons of the posterior central brain (*N* = 8). Based on these cell counts, DATI- and CHA-positive cells (i.e., cells that cause rejection with RNAi) correspond to a modest 4% of the total neurons in the central brain. Besides reducing the complexity of the neural circuit required for acceptance, these experiments revealed that *dati* is not required to determine cholinergic cell identity. Instead, *dati* appears to specify a subtype of neuronal identity that is presumably shared by neurons that express different neurotransmitters.

### Mapping Brain Regions Where *dati* Is Required to Generate Acceptance Reveals Discrete Brain Foci

**Figure 4:**
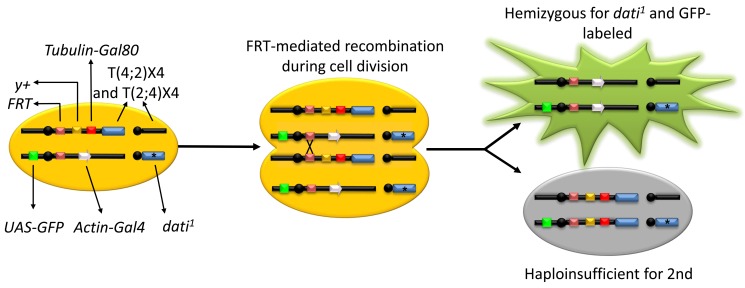
The FYT system. The FYT system works in a manner similar to traditional MARCM, but with a reciprocal translocation bringing the fourth chromosome to the end of the second chromosome containing a centromeric FRT site. In this system, each cell has a heat shock-inducible source of Flippase on the X chromosome (not shown), plus two copies of the fourth chromosome (blue boxes), one carrying a mutant allele *dati^1^* (asterisk) and the other carrying a wild-type copy translocated to the second chromosome. The second chromosome with the appended fourth chromosome contains an FRT site for somatic recombination, a *y*+ transgene to mark external tissues, and a ubiquitous source of the Gal4 repressor, Gal80 (red box). The homologous second chromosome carries an FRT site and a ubiquitous source of Gal4 (grey arrow) driving UAS-GFP (green box), which is repressed by Gal80. The induction of Flippase by heat shock (not shown) in a dividing cell removes the Gal80 repressor and generates two types of daughter cells. One that expresses GFP (green cell) and is hemizygous for a fourth chromosome bearing the mutation of interest, and another cell that dies due to aneuploidy (grey cell) [23].

**Figure 5:**
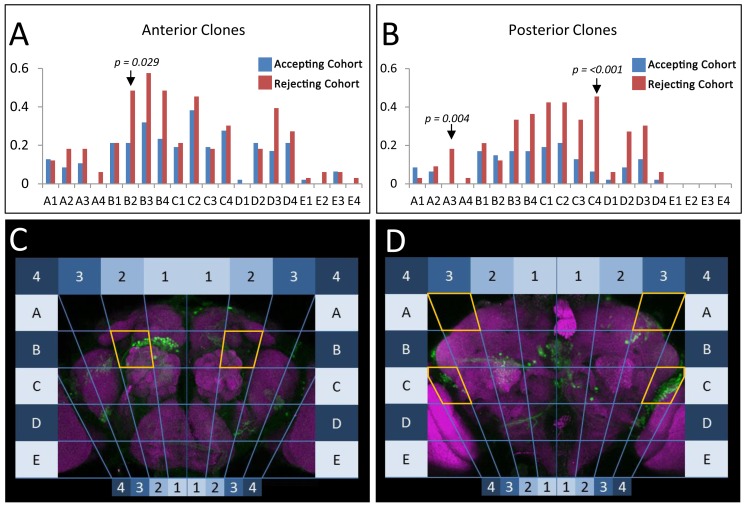
Mapping regions of the brain where *dati* is required for normal acceptance using the GAF/FYT system. (A and B) Frequency of marked *dati^1^* clones in the brains of rejecting (red) and accepting (blue) females. Analysis of brains is divided into anterior (A) and posterior (B), and subdivided by regions corresponding to the maps seen in (C, D). The statistical significance of differences between accepting and rejecting clones was evaluated by the Fisher's exact test. The p values are indicated in the figure. Brain images are maximum intensity projections of confocal stacks rendered in Zen 2009.

To determine the brain regions that mediate acceptance, we performed a clonal analysis using a new genetic tool we developed that allows for the systematic and efficient generation of somatic clones of fourth chromosome mutants, named the FYT system (Figure 4) [23]. In these experiments, we randomly removed *dati* in different positions in the brain, tested whether females accepted or rejected males, and located the position of each clone within a grid that divides the brain in 80 sectors (Figure 5A–D). By compiling a collection of 491 clones in the brain of females that either produce acceptance or double rejection (i.e., rejection in 2 consecutive days), it becomes clear that some regions in the brain produce significant deficits in acceptance while others do not. In the anterior brain, a single statistically significant region was identified in anterior sector B2 (AntB2, *p* = 0.029, Figure 5A). In the posterior brain, two regions stood out as highly significant (PosA3, *p* = 0.004 and PosC4, *p*≤0.001) (Figure 5B).

The anterior region AntB2 encompasses the first focus identified for female acceptance behavior using gynandromorphs [19] and also a region populated by extensively characterized local neurons (LNs) that express Sex lethal [50],[51]. The posterior region PosA3 is located in the posterior superior lateral protocerebrum (pslpr) immediately above the lateral horn. In contrast, PosC4 spans over the ventral part of the lateral horn, the edge of the posterior inferior lateral protocerebrum (pilpr), and posterior lateral protocerbrum (plpr) (Figure 5D). Together, these results show that *dati* is required in discrete neurons along a known olfactory path [52],[53], which involves second-order olfactory neurons and also third-order neurons located around the lateral horn. Interestingly, the ventral lateral horn has been recently identified as the region that processes pheromones [54],[55]. In contrast, PosA3 appears to be a novel focus implicated in female receptivity.

### Rejection Foci Contain Few *dati*-Positive, Cholinergic Neurons

**Figure 6:**
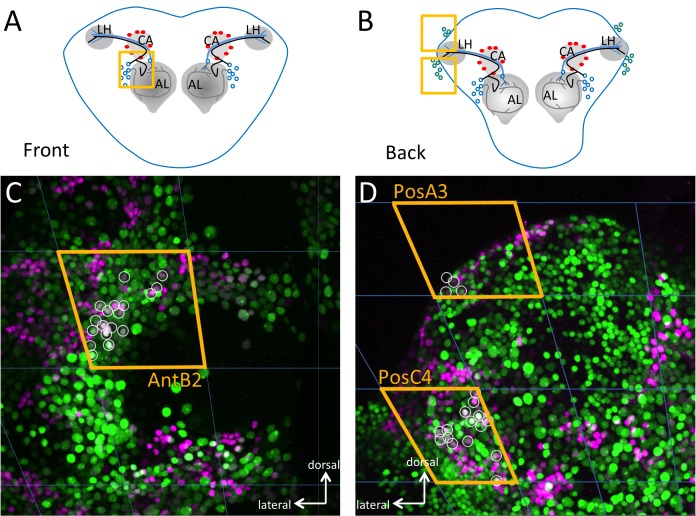
Detailed view of DATI/CHA double-positive cells in the three foci implicated in female acceptance as revealed by clonal analysis. (A) A schematic frontal view of the brain where the antennal lobes (AL), the calyx (CA), and the lateral horn (LH) are indicated. The square indicates the position of the focus AntB2 indicated in (C). The trajectories of two projection neurons towards the lateral horn (black and blue line) and a projection of an LN are shown (grey line). The cell bodies around the antennal lobe are depicted as empty circles and the Kenyon cells as red circles. (B) Schematic rear view of the brain with the same three neuropiles and the Kenyon cells indicated in (A), the position of the foci PosA3 (upper square) and PosC4 (lower square). Cell bodies in the lateral horn are shown as empty circles. (C) Frontal maximum intensity projection of the antennal lobes as depicted in (A) with DATI protein stained in magenta, *Cha*-Gal4 UAS-mCherry.NLS in green, and the overlap between the two in light magenta or white (small circles). To determine the overlaps in a single image, the beginning and end of the double-labeled nuclei were identified in the z-stacks and a circle was drawn around the nucleus of the maximum intensity projections. In most cases the overlaps can be seen in the maximum intensity projection, but in a few cases this is not evident. The lateral and dorsal orientation of the neuropiles in (C) and (D) are indicated in the figure. Quantification of cell numbers and sample sizes are indicated in text.

To narrow down the position of the neurons in each sector, we analyzed the neurons that express CHA and DATI within these regions. In the anterior brain, within the region AntB2, we can discern 13.8±2 neurons per hemi-antennal lobe (*N* = 16, Figure 6A and C). The posterior brain regions PosA3 and PosC4 that produced the most significant acceptance deficits also have very few DATI CHA neurons. Indeed, in these two regions we can identify 16.82±2.4 neurons that are positive for DATI and CHA (*N* = 15, Figure 6B and D). In the PosA3 sector (pslpr), we found 3.64±1.18 neurons (Figure 6D), and in the ventral lateral horn and posterior inferior lateral protocebrum (pilpr), there are 13.17±2.52 DATI CHA-positive neurons (*N* = 15). These results suggest that a strikingly small number of DATI CHA neurons are essential for female acceptance.

### *dati* Is Required to Generate a Subtype of Cholinergic Neurons

**Figure 7:**
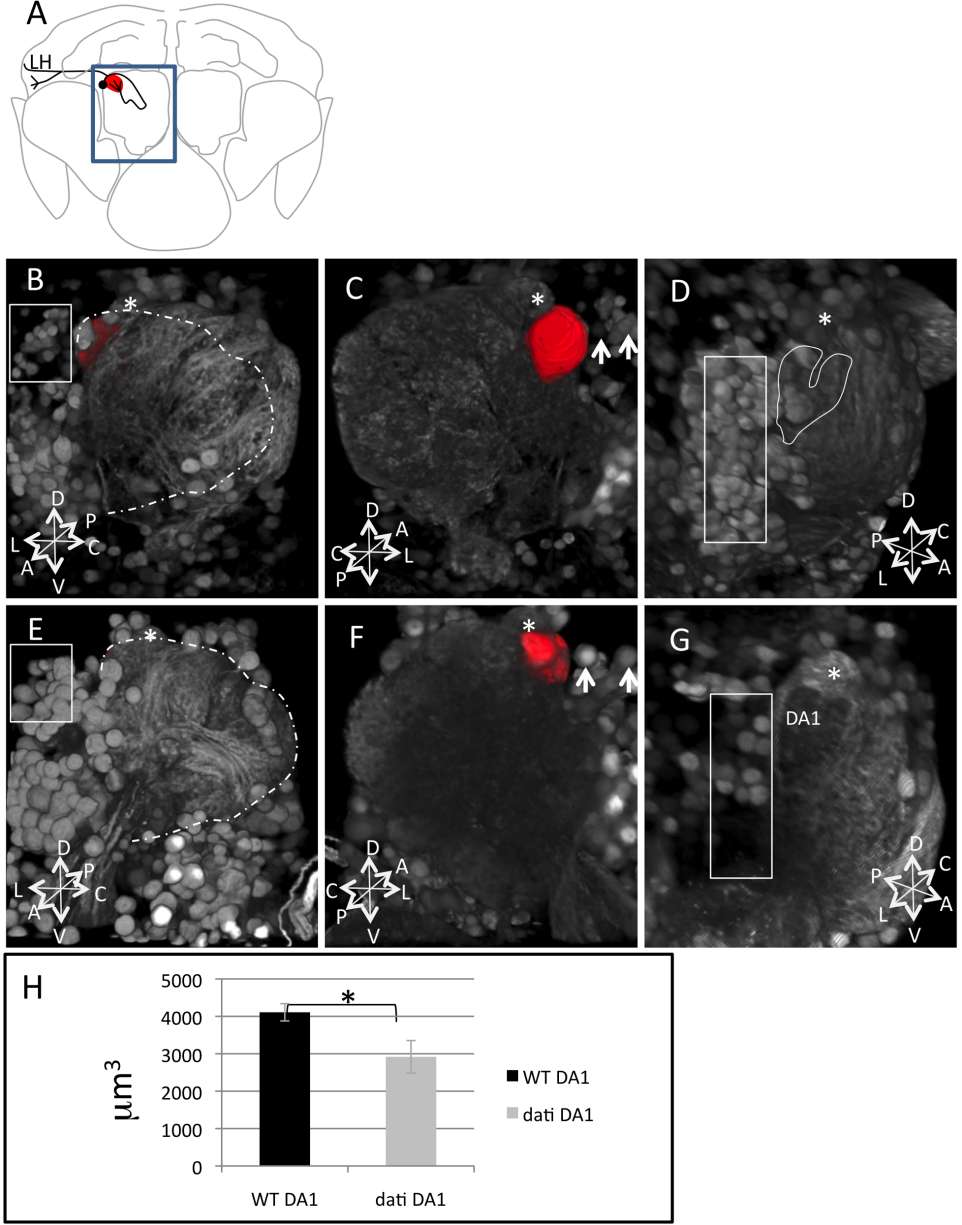
Homozygosity for *dati^1^* causes loss of cholinergic projection neurons in the antennal lobe. (A) The location of the antennal lobe in the brain (blue square). The cell body of a single lateral cholinergic projection neuron is indicated (black circle). Some cholinergic neurons in this position connect to the antennal lobe neuropile, which include the glomerulus DA1 (red, also shown in B and C) and the lateral horn (line in black). (B) Frontal view of a wild-type antennal lobe with the glomerulus DA1 highlighted in red by 3D segmentation and the glomerulus DL3 (asterisk). The dashed lines indicate the area occupied by the neuropile of the mutant in (E) and the square lateral neurons expressing CHA. (C) The same antennal lobe in (B) viewed from the brain outwards. The DA1 glomerulus (red) and the glomerulus DL3 (asterisk) are indicated. Arrows point to lateral neurons. (D) Side view of a wild-type antennal lobe. The square indicates lateral neurons expressing CHA, the asterisk the position of glomerulus DL3 (not visible from this angle), and the heart shape outlines the position of DA1 shown in (G). (E) Frontal view of a *dati* mutant brain, highlighting the antennal neuropile (dashed lines), the position of the glomerulus DL3 (asterisk), the glomerulus DA1 (also segmented in red to the left of DL3, but not visible from this angle in the 3D rendering), and the lateral neurons (square). Note that the antennal lobe neuropile in (E) is smaller than in (B), which indicates defects in innervation. Also note that the neurons in the square in (B) are smaller than those in (C). (F) Mutant antennal lobe in (E) viewed from the brain outwards. Asterisks indicate glomerulus DL3, red indicates glomerulus DA1, and arrows point to lateral neurons. Note that the size of DA1 in (F) is smaller than in (C) and that the lateral neurons are larger in (F) than in (C) (arrows). (G) Side view slightly from the top of another antennal lobe mutant for *dati* ^1^. The DL3 (asterisk), DA1 (red), and lateral neurons (square) are indicated. Note that the neuropile where DA1 is indicated in (G) appears darker than other parts of the antennal lobe and also darker than the corresponding region in (D) (heart-shaped outline), indicating that this region is less dense. Also note that the density of lateral neurons in (G) (square) is smaller than in (D) (square). Images are 3D projections of confocal stacks rendered in Fiji. (H) Quantification of DA1 volume in the wild type and mutant. Error bars indicate SEM. * indicate *p*<0.05, and the samples sizes are Canton-S, *N* = 6 and *dati* ^1^, *N* = 6. The coordinates P (posterior), A (anterior), D (dorsal), V (ventral), L (Lateral), and C (Central) are indicated.

Because we had observed that the removal of *dati* in olfactory neurons in the region AntB2 impairs female acceptance and *dati* is required in the specification of late born neurons [35], we expected that the mutant might fail to specify a neuronal subtype DATI CHA. To begin addressing this issue, we compared the GFP expression patterns of *Cha*-Gal4 in wild-type and *dati^1^* homozygotes (Figure 7A–G). These experiments revealed severe abnormalities in the cholinergic tracts of the antennal lobes (Figure 7B–G). A closer examination reveals that the population of dorsal lateral neurons in the region AntB2 are either reduced or transformed to cholinergic neuronal types with a distinct morphology than those normally found in this region (Figure 7C and F). These transformations within antennal lobe neurons affect several glomeruli, which include DA1, the target of the male pheromone cis-vaccenyl acetate (cVA) (Figure 7D–H) [56].

The experiments above revealed that the loss of *dati* disrupts olfactory glomeruli. To test whether these disruptions lead to olfactory deficits, we assayed the performance of *dati* mutant females in a T-Maze in which flies are tested for moving away or towards an aversive odor. In this test, only 3% of the Canton-S flies (1 out of 30) moved towards the aversive odor compared to 32% of the *dati* mutant females (11 out of 34), indicating that olfactory behavior is indeed impaired in *dati* mutants (wild type versus *dati*, *p* = 0.001).

**Figure 8:**
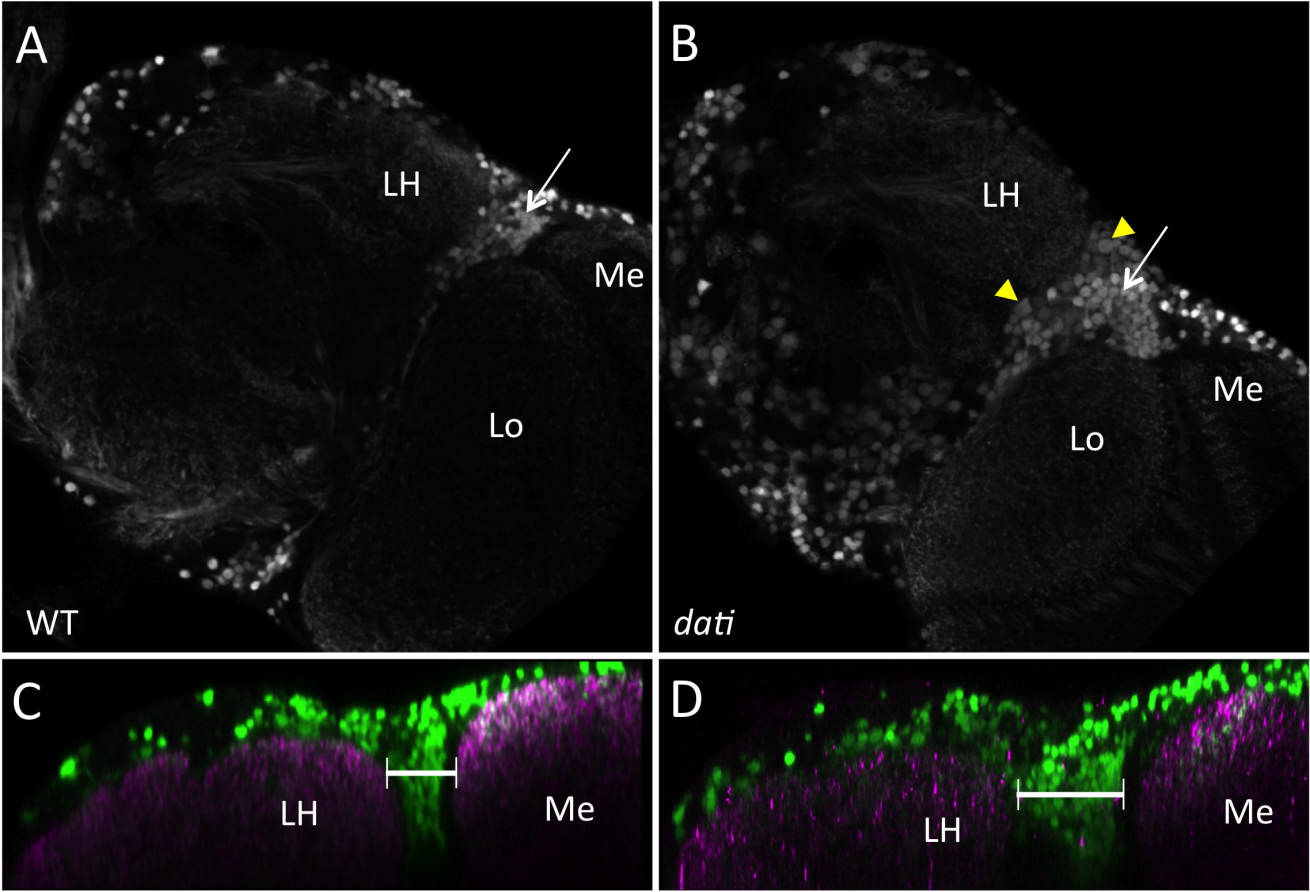
Homozygosity for *dati^1^* causes abnormal distribution of cholinergic neurons and improper innervation of the lateral horn. (A) Wild-type female brain expressing GFP under the control of *Cha*-Gal4. The image is a single frontal confocal slice at the level of the lateral horn. The lateral horn (LH), the medulla (Me) and lobula (Lo) are indicated. Note the density of cholinergic neurons in the region between the lateral horn and optic lobe (arrow). (B) Frontal confocal slice of a *dati* ^1^ female brain expressing GFP under the control of *Cha*-Gal4 in approximately the same position shown in (A). Note increased numbers of CHA+ cells (arrow) and enlarged cells (yellow arrowheads). (C, D) Orthogonal view of the region shown in (A, B). CHA+ cells are labeled in green, and the neuropile is labeled with nc82 antibody (magenta). (C) Wild-type. (D) *dati* ^1^ mutant female. Note again the excess of CHA+ cells in (D) compared to (C, brackets). Image rendering in orthogonal views in (C, D) were done in Image J.

In the lateral horn, the loss of *dati* leads to a reduction of approximately 10% of the lateral horn neuropile area (Figure 8C and D; *dati*, *N* = 8; WT, *N* = 10) and the cholinergic projections from the antennal lobe towards the lateral horn are also affected (Figure S4). Like in the antennal lobe, we note the presence of larger neurons in the lateral horn of *dati* mutants, which are not present in the wild type (Figure 8A and B). Furthermore, there are more CHA-positive cells around the lateral horn, suggesting that in the absence of *dati* some neuronal precursors can proliferate to later assume a cholinergic fate or, alternatively, that in the absence of *dati* some cells assume a cholinergic fate (Figure 8A–D). Together, these results show that *dati* is required in postmitotic neurons as well as in the precursors of these cells.

### Nuclear Bar Coding Reveals That *DATI CHA* Neurons Mediate Short- and Long-Range Connections

**Figure 9:**
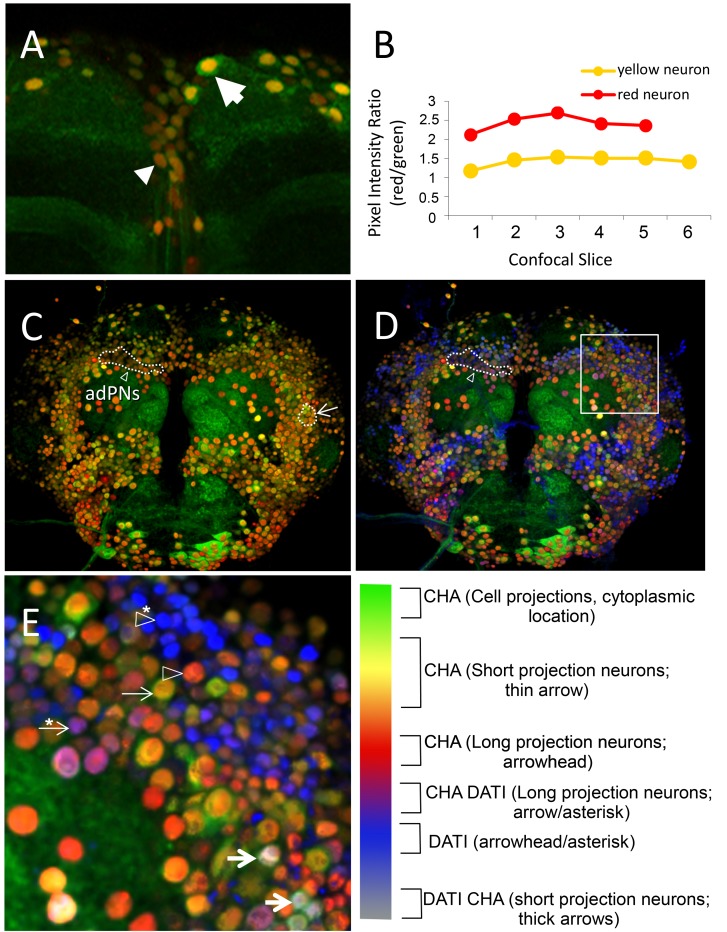
*dati* is expressed in neurons that have small and large volumes. (A) Maximum intensity projection of a confocal stack showing an antibody staining of a wild-type brain co-expressing nuclear RFP (red) and GFP-S65T (green) under *Cha*-Gal4 control. Neurons with large volumes (i.e., with long-range projections) appear with a gradation of dark orange to red (arrowhead; descending neuron), whereas neurons with small volumes (i.e., with short-range projections) appear as yellow to light orange (arrow). (B) Ratios of pixel intensity of the RFP and GFP channels of individual nucleus of the red and yellow neurons seen in (A) across all confocal slices encompassing their nuclei. Note the higher ratio of red/green from the red neuron with long processes, compared to the yellow neuron across each slice, indicating that the levels of GFP in the nuclei of long-range neurons are lower than in short-range neurons. Compare with results of the control experiment shown in Figure S4. (C) Overall view of the central brain. Anterior–dorsal projection neurons appear as dark red (adPNs, dotted line indicated by arrow), consistent with their long projections. LNs appear as light orange/yellow (dotted line indicated by arrowhead). (D) Superimposition of the channel detecting signal for anti-DATI staining (blue) to the image in (C). Note that adPNs appear in purple, indicating that these cells with long projections also express DATI (dotted line indicated by arrowhead). (E) High magnification of AntB2 region (box in D). DATI is expressed in cells with both small and large volumes, as indicated by color bar legend shown on the right. Neuron cell types are indicated by arrows and arrowheads, as explained in color bar legend. 3D image rendering was done in Image J.

From the previous experiments, we found evidence that *dati* specifies a subpopulation of cholinergic neurons that project into the antennal olfactory glomeruli. Olfactory neurons in the antennal lobe descend from few neuroblast lineages that generate remarkably different neurons within and across lineages [50], and it has been suggested that morphologically different neurons are dedicated to specific neurocomputations [57]. This heterogeneity has been traditionally investigated in great detail in clones of single or few neurons using Gal4 drivers that reveal discrete neuronal populations [58]–[60]. However, we are often confronted with the opposite problem, which is to estimate whether a selected neuronal population makes simple, complex, or both simple and complex connections when a discrete Gal4 driver for these neurons is not available. This distinction is important to determine whether *dati* intrinsically modifies cell shapes or other aspects of neuronal physiology [61]. To that end, we developed a simple system of nuclear bar coding that distinguishes different DATI CHA neurons by color. Nuclear Bar Coding (NBC) consists of labeling nuclei of neurons with small or large volumes with different colors by expressing a localized nuclear RFP (mCherry.NLS) and GFP-S65T (nuclear and cytoplasmic) under the control of a Gal4 driver (in this case *Cha*-Gal4). Cells expressing the two fluorescent proteins from the same promoter are expected to be produced and degraded at comparable rates and result in nuclei with an overlay of two colors (Figure S5) [62],[63]. Assuming that these two proteins are not subject to a different regulation, the overlay of two colors should vary depending on the cellular volume. In cells with long or more intricate processes, GFP-S65T should be expected to fill up the cellular processes and shift the overlay of the two signals in the cell bodies towards that of the localized nuclear fluorescence (i.e., red color from RFP). Evidence for this shift was obtained in comparisons between cells with short and long cell processes (Figure 9A–C). Conversely, when both GFP and RFP are targeted to the nucleus, the shifts of nuclear bar coding are abolished (Figure S5). If, to this simple bar coding, we add a third color that detects DATI-positive cells (Figure 9D), then we can globally assess whether *dati* cholinergic neurons have simple or more complex projections. NBC allowed us to easily identify the descending neurons (Figure 9A), as well as long projection neurons located immediately above the antennal lobe, known as anterior–dorsal projection neurons (adPNs; Figure 9C,D), and LNs imbedded in antennal lateral neurons (Figure 9C–E). In addition, the NBC method reveals that the DATI CHA neurons within the region AntB2 make both short and long connections (Figure 9C–E). Thus, we conclude that *dati* does not specify only one type of cell shape, like other transcription factors that specify particular neurons [61].

## Discussion

### *dati* Encodes a Conserved ZNF Transcription Factor Related to Rotund/Squeeze and ZNF384 Required for Female Decision Making and Locomotion

Here we described DATI, a zinc finger transcription factor related to the Drosophila Rotund and Squeeze and the vertebrate ZNF384, one of the three genes known to be involved in acute lymphoblastic leukemia (ALL) [64],[65]. A survey of the sequences related to *dati* suggests that it descends from a *Krüppel/rotund* prototype present in cnidarians (e.g., Nematostella, gb|ABAV01025004.1|). Later this prototype evolved to become the rotund-like found in nematodes (e.g., *C. elegans*, Lin29) and mollusks (e.g., *M. galloprovincialis*, gb|GAEN01018610.1|) and was inherited by both vertebrates and invertebrates. Due to its similarity with Lin29, *dati* was previously referred to as Dmel/Lin29. However, orthology tests show that the ortholog of the *C. elegans* Lin29 is *rotund*, not *dati*. The first true ortholog of *dati* is found in marine arthropods (e.g., *Daphnia pulex*, Dpdati, gb|ACJG01001740.1|), which appeared in the Cambrian some 540 Mya [66].

Like its vertebrate homolog, *dati* is expressed in the nervous system and required for stem cell development [35],[67]–[69]. During embryogenesis, *dati* is one of the last genes to be activated in a serial activation of transcription factors that determines the identity of specific neuronal lineages in the ventral nerve cord [35]. The present study shows that *dati* is later required to specify regions of the central brain required for appropriate female acceptance.

*dati* mutant flies are moderately uncoordinated and almost invariably reject male courtship (Figure S1C and Figure 2). This rejection is so intense and persistent that it does not seem to be due to the mere loss of single sensory modalities, which inhibit but do not abolish acceptance [36]. Because of this strong rejection, we expected that *dati* might impair either more than one path required to generate acceptance in the brain or an area in which sensory information converges. In addition, we also tested if the locomotor and decision-making defects were associated or separable.

### Locomotor Defects Are Separable From the Inability to Make Decisions

The mapping of foci by clonal analyses revealed individuals with clones that exhibited rejection but not locomotor defects (unpublished data). Conversely, we also found individuals with locomotor defects that were perfectly capable of accepting courtship and mating properly (unpublished data). Further evidence that locomotion and female behavior are separable was obtained in the experiments in which *dati* was knocked down in neurons that express different neurotransmitters (Figure 2). In this case, we found that none of the four drivers used (*Ddc*, *Gad*, *ple*, and *Cha*-Gal4) produced locomotor defects like those observed using either a ubiquitous driver or the neuronal driver *elav*-Gal4, but the removal of *dati* in CHA neurons resulted in strong female behavior deficits. Thus, we conclude that the locomotor defects and female acceptance map to different brain regions and distinct cells that express specific neurotransmitters.

### DATI Adds an Additional Layer to the Identity of Cholinergic Neurons That Is Shared by Noncholinergic Neurons

Our results suggest *dati* has two roles in the nervous system—one developmental and another constitutive—both affecting female behavior. The over/underproliferation of cholinergic neurons in *dati* homozygotes suggests a requirement in neuronal precursors, which is consistent with the previous study that showed *dati* is transiently expressed in developing ganglion mother cells [35]. However, there is a requirement in neurons, as the courtship behavioral phenotype is recapitulated when *dati* is removed in postmitotic neurons. Further evidence for this requirement in adult neurons is the fact that *dati* is indeed expressed in neurons well into adulthood, and in fact, we identified a small group of neurons that only initiates expression of *dati* in adult neurons (unpublished data). Together these results suggest that *dati* may be required to maintain a neuronal identity. Because not all *dati*-positive neurons are cholinergic, and vice versa, it is unlikely that its primary role would be to determine the expression of this neurotransmitter. The Nuclear Bar Coding analysis suggests that *dati* does not evidently define any specific cell morphology either. We speculate that *dati* specifies a type of neuronal identity that allows neurons to respond to neurotransmitters that other cholinergic neurons without *dati* cannot. In this scenario, it is easy to see that removing *dati* from mature neurons would deprive them from the appropriate receptor(s) needed to receive input from their synaptic partners, and consequently silence female receptivity. Future tests should resolve whether *dati* indeed regulates channels/receptors to generate courtship acceptance.

### The Regions Where *dati* Is Required Agree with Previous Mapping and Suggest the Existence of a Core Circuit for Female Decision Making

Different mutants and experimental approaches, including gynanders, *spinster* mosaics, mapping of cVA processing neurons, and the use of *dati* mosaics, here have identified some common and other distinct foci for female decision making. For instance, the first focus AntB2 that we identified maps to Sp11, the first brain region identified for female acceptance using mosaic gynandromorphs [19]. AntB2 also maps within the Spin-D site identified by mosaics of *spinster* [18], a gene also required for female behavior. In addition, the two other highly significant regions, PosC4 and PosA3, flank the lateral horn, and we note that the focus PosC4 co-maps with regions previously implicated in pheromonal processing in the female brain [52],[54]. Notably, the lateral horn may have a larger role in sensory integration, as it receives projections from centers that process visual and mechanosensory information [52]. Thus, the picture that emerges from previous work and the present study suggests that female decision making in *Drosophila* is modulated by a core circuit involving the antennal lobe and the lateral horn. However, we note that there are regions with ratios of acceptance and rejection that intuitively may appear to be relevant but that failed to reach statistical significance. In particular, there are three regions in the anterior brain (AntB3, AntB4, and AntD3) and seven regions in the posterior brain (PosB3, PosB4, PosC1, PosC2, PosC3, PosD2, and PosD3). We believe that these regions are unlikely foci for female receptivity, as our sample had resolution to identify the great significance of a relatively small focus like PosA3. Also, a similar study that analyzed a larger sample of Spinster foci for female receptivity also found brain regions that did not reach statistical significance but had ratios that could be intuitively interpreted as almost significant like ours. Like us, these authors disregarded these data as significant [18].

### *dati*'s Requirement in Few Excitatory Neurons in Three Discrete Brain Foci Reveals a Simple, Yet Fundamental, Mechanism of Female Decision Making in Drosophila

Besides providing the locations where courtship acceptance decisions are generated in the brain and the type of neurotransmitter involved, our results also reveal a significant neural mechanism at play. The DATI-CHA neurons mapped in the antennal lobe correspond to a subset of extensively studied cholinergic population known as the excitatory dorsal lateral Projection Neurons (ePNs) and excitatory lateral neurons (eLNs) [70]–[75]. The central role of excitatory cholinergic neurons revealed by our study and the localization of a region where sensory information is integrated constitute a nearly perfect cellular and molecular representation of the “Summation Hypothesis,” elaborated by Manning and others several decades ago based on behavioral inference [38],[76],[77]. This hypothesis states that acceptance of courtship involves the convergence of multiple excitatory stimulations provided by different sensory modalities until the stimulation reaches a critical threshold point that generates acceptance [76]. Most importantly, the Summation Hypothesis predicts that the two opposite female responses (i.e., rejection or acceptance) are not the result of opposing neural activities (e.g., excitation and inhibition) but rather the result of two different levels of excitation. Until now, there was no molecular and cellular evidence in support of this prediction. In this regard, our results are in agreement with this prediction, as the absence or presence of DATI in an excitatory circuit generates either complete rejection or overwhelming acceptance, respectively.

Corroborating our results, recent findings show that pheromone processing is not subject to the inhibitory mechanisms that apply to the processing of other odors [78]. Taken altogether, our results suggest that few dozen excitatory neurons converging in as few as three brain foci make the core components to generate a mating decision in *Drosophila*. Given that *dati*-related genes are present in a wide variety of organisms, it is likely that their common ancestor had the same or a similar mechanism of female acceptance.

## Supporting Information

Figure S1 **Embryonic and larval expression of** ***dati*** **.** (A) Antibody staining of a wild-type late stage embryo for DATI (green) and ELAV (magenta). Note the presence of DATI in neurons of each hemisegment of the ventral nerve cord. Image is from a single slice of a confocal image stack. (B) Antibody staining of a wild-type L3 larval brain for DATI (gray). (C) Adult female brain stained with anti-DATI (green). The images are maximum intensity projections of confocal stacks.
(TIF)

Figure S2 **Quantification of discrete responses to male courtship displayed by** ***dati*** **mutant females versus wild-type females.** Female response to male courtship was quantified for 10 min after initiation of courtship by wild-type males. Bars show BI of each discrete response type of control group (Canton-S females, dark bars) and experimental (*dati* homozygous females, light bars) (see Materials and Methods for details). *dati* females are capable of displaying the same array of rejection behaviors to courtship as wild-type females (i.e., fleeing, kicking, extruding ovipositor, jumping, and flicking wings). Compared to wild-type females, *dati* females spend more time kicking males. In contrast, *dati* females spend significantly less time standing still, which is considered an accepting behavior displayed by wild-type females after being courted for some time. The statistical significance of differences was evaluated by the Mann–Whitney *U* test (***p<0.001; **p<0.01), and error bars represent ±SEM. The sample sizes are Canton-S, *N* = 12 and *dati* ^1^, *N* = 10.
(TIF)

Figure S3 **Cell counts of neurons expressing DATI and both DATI and CHA.** Cell counts of two different classes of neurons in Canton-S adult flies: DATI CHA double-positive cells and DATI-only positive cells. Light grey bars represent counts from scans made from the front of the central brain; black bars represent counts from scans made from the rear of the central brain. Automated cell counts were performed in Fiji as described in Materials and Methods.
(TIF)

Figure S4 ***dati*** **mutants exhibit defects in the trajectory of projection neurons.** (A) A wild-type brain and (B) a *dati* ^1^ mutant brain viewed from the brain neuropile towards the rear surface of the brain. In both images, 3D-rendered images were superimposed to 3D segmentation of the major cholinergic tracts (magenta). The lateral horn (LH) is indicated by the dashed circle. (C and D) Isolated segmentations of the major cholinergic tracts of the brain shown in (A and B, respectively) viewed from the rear brain surface. Note the thickness and complexity of the cell projection coming from the antennal lobe in (C) (red bracket) and the thinner and ill-defined projections in *dati* ^1^mutants (D) (red bracket). The coordinates P (posterior), A (anterior), D (dorsal), V (ventral), L (lateral), and C (central) are indicated.
(TIF)

Figure S5 **Nuclear GFP and nuclear RFP have comparable rates of degradation.** Fluorescent signals of nuclear GFP and nuclear RFP driven by the same ubiquitin promoter in the adult brain were captured and quantified. (A) A high-magnification confocal slice of the antennal lobe. The GFP and RFP pixel intensity values were collected along the white arrow. (B) Greyscale view of GFP.NLS expression from (A). (C) Greyscale view of RFP.NLS expression from (A). (D) Quantification of signals captured along the line shown in (A). Note that the levels of RFP and GFP are similar across the intensity peaks and valleys in contrast to when nuclear bar coding is performed (Figure 9).
(TIF)

Movie S1 **Movie of a 2-d-old Canton-S female being courted by a Canton-S male.** Note that although the female flees to some degree, she does so slowly and eventually stops to fully take in the males courtship display, and eventually allows him to mount.
(MP4)

Movie S2 **Movie of a 2-d-old** ***dati*** **homozygous female being courted by a Canton-S male.** Note that the female flees quickly from the male, and when she is caught up to by the male, she engages in rejection behaviors such as kicking.
(MP4)

Movie S3 **Movie of a 7-d-old** ***dati*** **female being courted by a Canton-S male.** Note the more severe incoordination of this female in comparison to younger female shown in Movie S2.
(MP4)

Movie S4 **A 3D rendering of segmented images of the mushroom body of wild-type,** ***Cha*** **-Gal4 UAS-** ***dati*** **-RNAi and** ***dati^1^*** **females labeled with anti-FasII.** Labels for each genotype are shown in the movie. 3D rotations showing α (red), β (blue), and γ (yellow) lobes. Note that the γ lobe has a hammer-like shape in wild-type and *Cha*-Gal4<UAS-*dati*-RNAi females, whereas in *dati^1^* mutants the γ lobe has an accentuated curvature in the center. This morphological defect is quantified in Figure 3D.
(MP4)

Text S1 **Determination of the molecular limits of l(4)102CD^d2^.**
(TIF)

## Abbreviations

adPN: anterior–dorsal projection neuron
ALL: acute lymphoblastic leukemia
ANOVA: analysis of variance
BI: Behavioral Index
*Cha*: *Choline acetyltransferase*
CI: Courtship Index
CS: Canton-S
cVA: cis-vaccenyl acetate
*dati*: *datilógrafo*
*dcr*: *dicer*
*ddc*: *dopa decarboxylase*
*elav*: *embryonic lethal abnormal vision*
eLNs: excitatory lateral neurons
ePN: excitatory dorsal–lateral projection neurons
FRT: flippase recognition target
FYT: *FRT*-*yellow*-translocation
*Gad*: *Glutamic acid decarboxylase 1*
GFP: green fluorescent protein
h: hour
iPN: inhibitory projection neurons
LH: lateral horn
Lo: lobula
Me: medulla
min: minute
MYA: million years ago
NBC: nuclear bar coding
NLS: nuclear localization sequence
pilpr: posterior inferior lateral protocerebrum
*ple*: *pale*
plpr: posterior lateral protocerebrum
pslpr: posterior superior lateral protocerebrum
*repo*: *reversed polarity*
RFP: red fluorescent protein
*rn*: *rotund*
s: second
SEM: standard error of the mean
*spin*: *spinster*
*sqz*: *squeeze*
WT: wild-type
