# Supplementary figures and images for: The KRÜPPEL-Like Transcription Factor DATILÓGRAFO Is Required in Specific Cholinergic Neurons for Sexual Receptivity in Drosophila Females
Source: PLoS Biol. 2014 Oct 7;12(10):e1001964. doi: 10.1371/journal.pbio.1001964 (PMC4188565; doi:10.1371/journal.pbio.1001964)

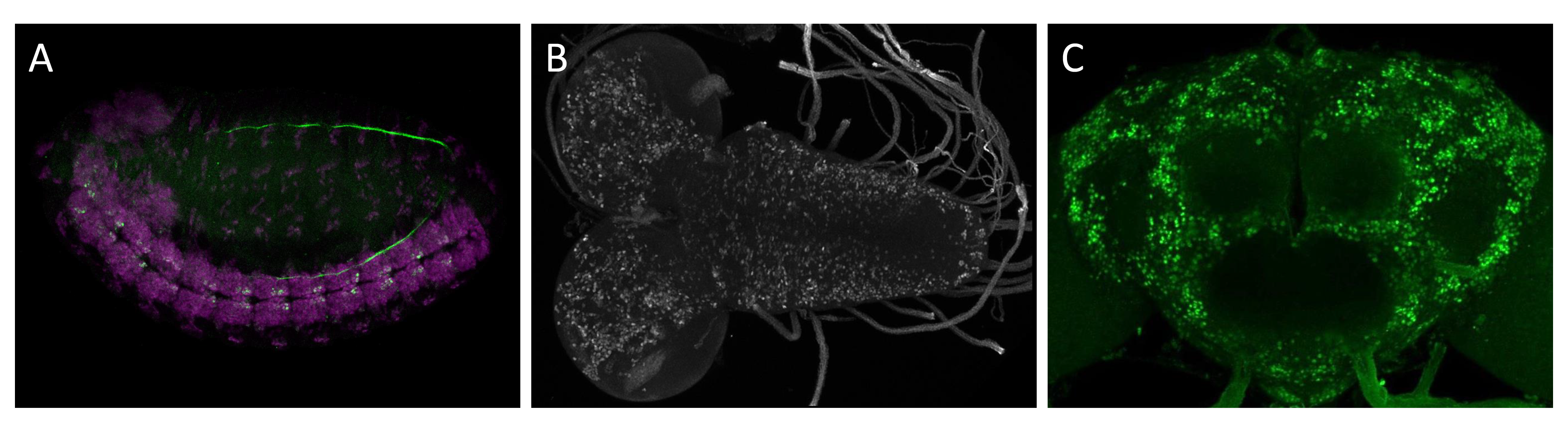

Supplement: Figure S1 — Embryonic and larval expression of dati . (A) Antibody staining of a wild-type late stage embryo for DATI (green) and ELAV (magenta). Note the presence of DATI in neurons of each hemisegment of the ventral nerve cord. Image is from a single slice of a confocal image stack. (B) Antibody staining of a wild-type L3 larval brain for DATI (gray). (C) Adult female brain stained with anti-DATI (green). The images are maximum intensity projections of confocal stacks. (TIF) [file pbio.1001964.s001.tif]

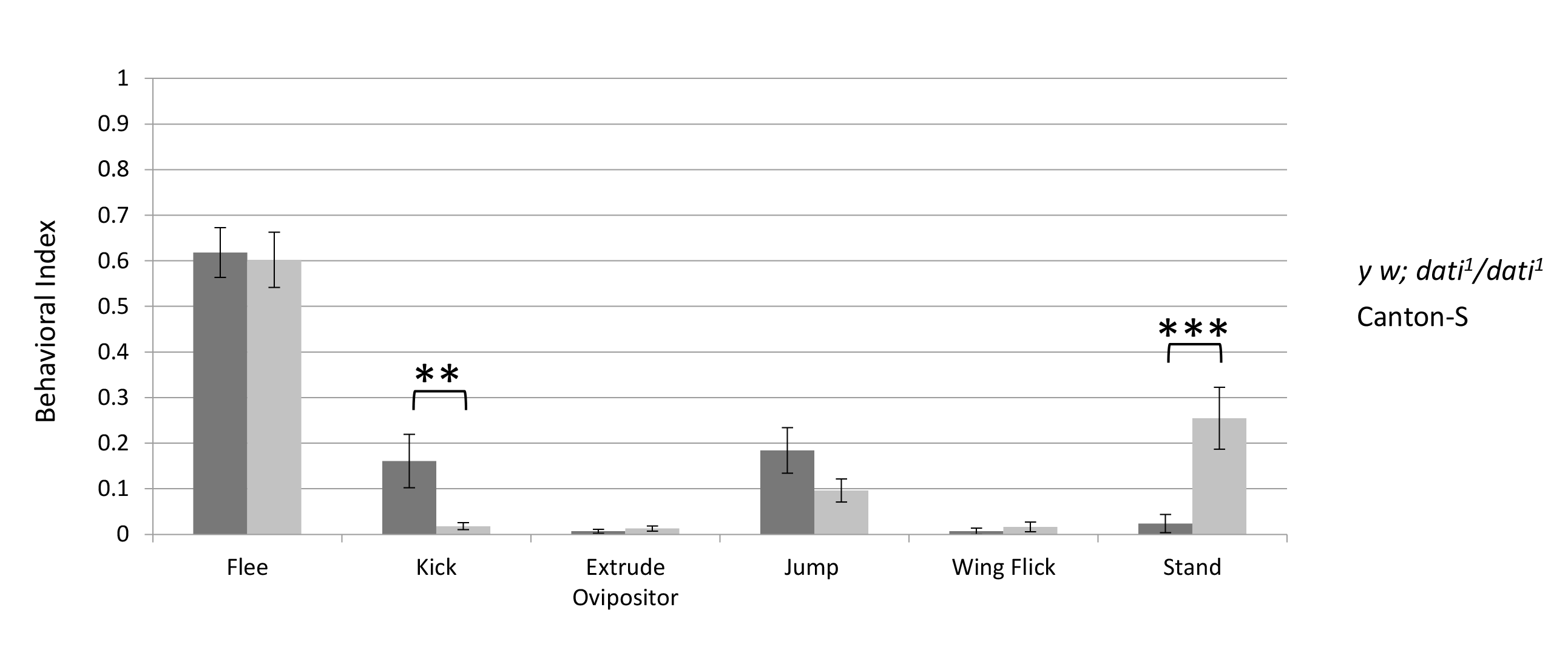

Supplement: Figure S2 — Quantification of discrete responses to male courtship displayed by dati mutant females versus wild-type females. Female response to male courtship was quantified for 10 min after initiation of courtship by wild-type males. Bars show BI of each discrete response type of control group (Canton-S females, dark bars) and experimental (dati homozygous females, light bars) (see Materials and Methods for details). dati females are capable of displaying the same array of rejection behaviors to courtship as wild-type females (i.e., fleeing, kicking, extruding ovipositor, jumping, and flicking wings). Compared to wild-type females, dati females spend more time kicking males. In contrast, dati females spend significantly less time standing still, which is considered an accepting behavior displayed by wild-type females after being courted for some time. The statistical significance of differences was evaluated by the Mann–Whitney U test (***p<0.001; **p<0.01), and error bars represent ±SEM. The sample sizes are Canton-S, N = 12 and dati 1, N = 10. (TIF) [file pbio.1001964.s002.tif]

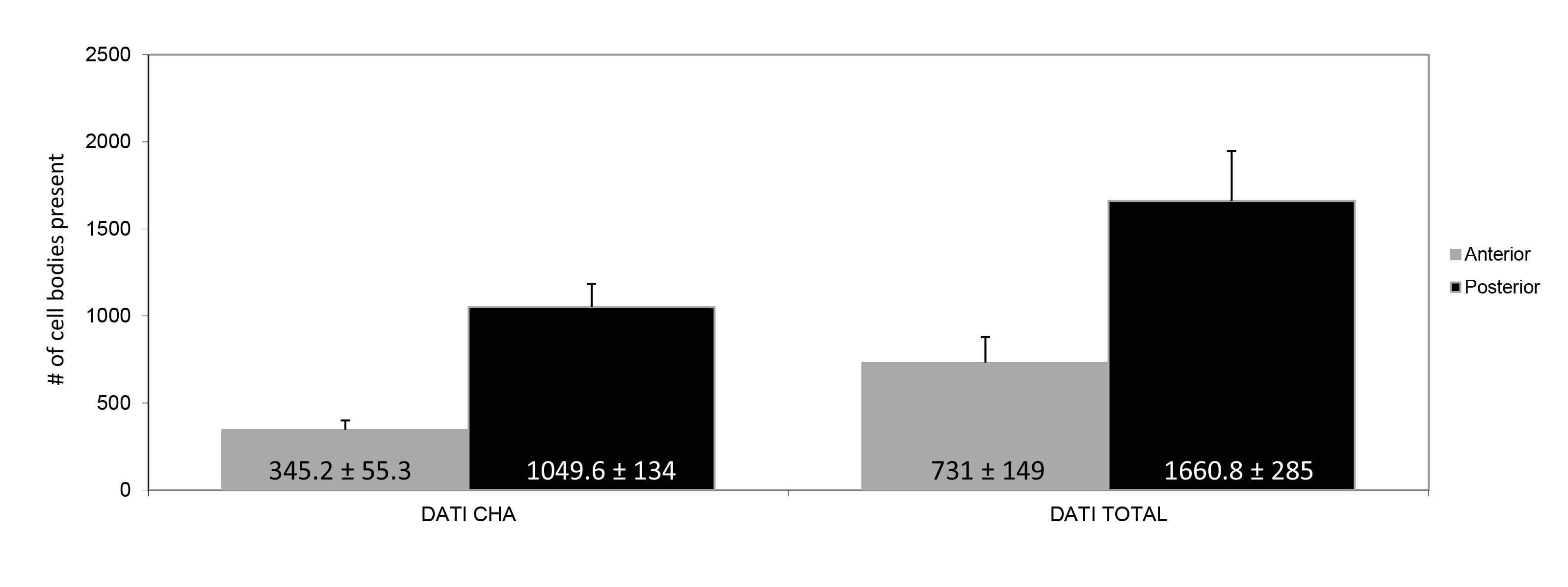

Supplement: Figure S3 — Cell counts of neurons expressing DATI and both DATI and CHA. Cell counts of two different classes of neurons in Canton-S adult flies: DATI CHA double-positive cells and DATI-only positive cells. Light grey bars represent counts from scans made from the front of the central brain; black bars represent counts from scans made from the rear of the central brain. Automated cell counts were performed in Fiji as described in Materials and Methods. (TIF) [file pbio.1001964.s003.tif]

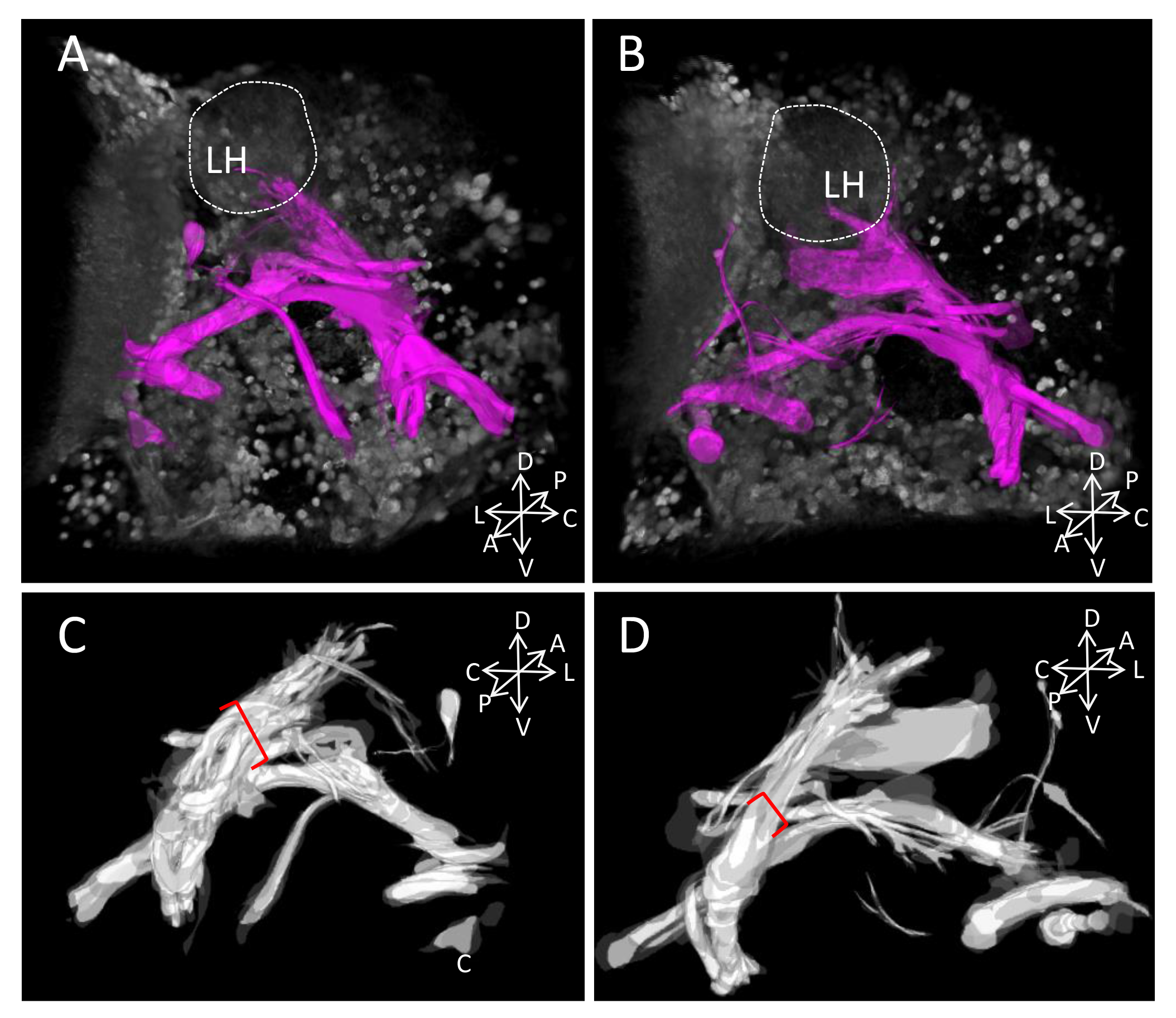

Supplement: Figure S4 — dati mutants exhibit defects in the trajectory of projection neurons. (A) A wild-type brain and (B) a dati 1 mutant brain viewed from the brain neuropile towards the rear surface of the brain. In both images, 3D-rendered images were superimposed to 3D segmentation of the major cholinergic tracts (magenta). The lateral horn (LH) is indicated by the dashed circle. (C and D) Isolated segmentations of the major cholinergic tracts of the brain shown in (A and B, respectively) viewed from the rear brain surface. Note the thickness and complexity of the cell projection coming from the antennal lobe in (C) (red bracket) and the thinner and ill-defined projections in dati 1mutants (D) (red bracket). The coordinates P (posterior), A (anterior), D (dorsal), V (ventral), L (lateral), and C (central) are indicated. (TIF) [file pbio.1001964.s004.tif]

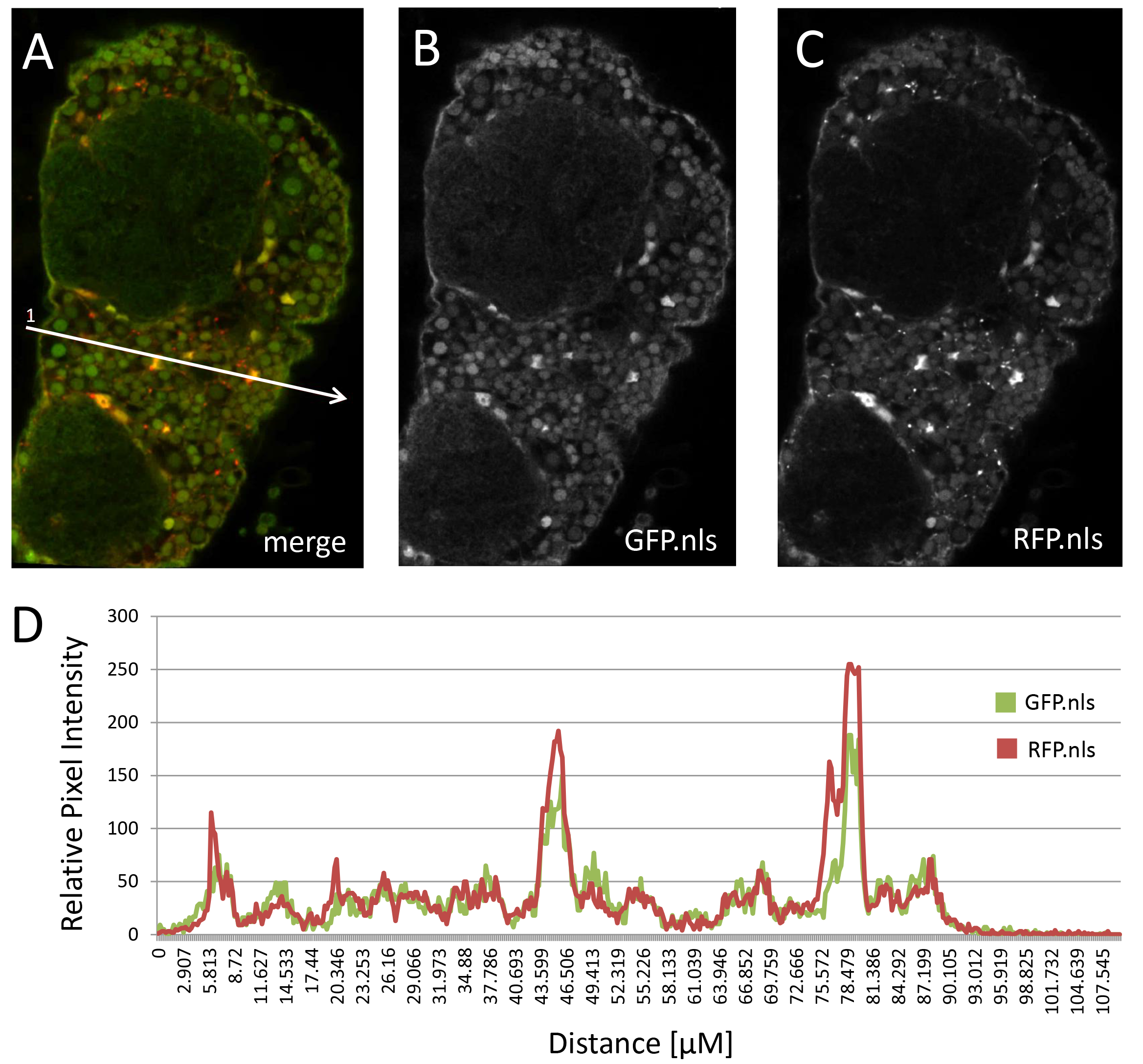

Supplement: Figure S5 — Nuclear GFP and nuclear RFP have comparable rates of degradation. Fluorescent signals of nuclear GFP and nuclear RFP driven by the same ubiquitin promoter in the adult brain were captured and quantified. (A) A high-magnification confocal slice of the antennal lobe. The GFP and RFP pixel intensity values were collected along the white arrow. (B) Greyscale view of GFP.NLS expression from (A). (C) Greyscale view of RFP.NLS expression from (A). (D) Quantification of signals captured along the line shown in (A). Note that the levels of RFP and GFP are similar across the intensity peaks and valleys in contrast to when nuclear bar coding is performed (Figure 9). (TIF) [file pbio.1001964.s005.tif]

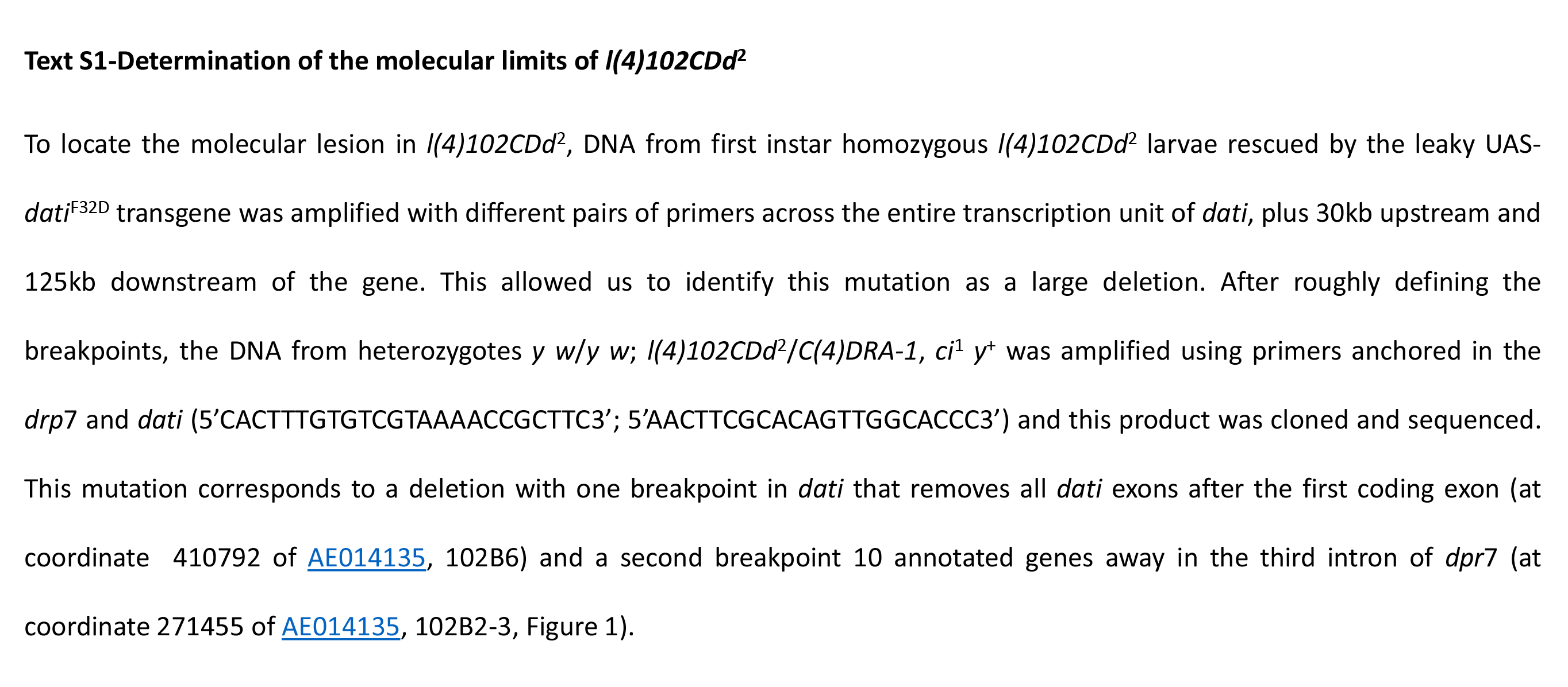

Supplement: Text S1 — Determination of the molecular limits of l(4)102CDd2. (TIF) [file pbio.1001964.s010.tif]
